# High perceived isolation and reduced social support affect headache impact levels in migraine after the Covid-19 outbreak: A cross sectional survey on chronic and episodic patients

**DOI:** 10.1177/03331024211027568

**Published:** 2021-07-13

**Authors:** Chiara Cerami, Chiara Crespi, Sara Bottiroli, Gaia Chiara Santi, Grazia Sances, Marta Allena, Tomaso Vecchi, Cristina Tassorelli

**Affiliations:** 1 Scuola Universitaria Superiore IUSS Pavia, Pavia, Italy; 2 Cognitive Computational Neuroscience Research Unit, IRCCS Mondino Foundation, Pavia, Italy; 3 Department of Brain and Behavioral Sciences, 19001 University of Pavia, University of Pavia, Pavia, Italy; 4 Faculty of Law, Giustino Fortunato University, Benevento, Italy; 5 Headache Science and Neurorehabilitation Centre, IRCCS Mondino Foundation, Pavia, Italy; 6 Cognitive Psychology Research Center, IRCCS Mondino Foundation, Pavia, Italy

**Keywords:** Covid-19, chronic migraine, episodic migraine, headache impact, loneliness, perceived isolation

## Abstract

**Background:**

Psychosocial variables are key factors influencing psycho-physical equilibrium in migraine patients. Social isolation and vulnerability to stressors may prevent efficient psychological adjustment negatively affecting adaptation to life changes, as that imposed during Covid-19 lockdown. Here, we explored psychosocial dimensions and changes in clinical condition during Covid-19 lockdown in migraine patients, with regard to migraine type and headache impact.

**Methods:**

Sixty-four migraine patients (32 episodic and 32 chronic) and 64 healthy control subjects were included in a case-control cross-sectional study. A two-step clustering procedure split patients into two clusters, based on the Headache Impact Test. Perceived global distress, loneliness, empathy, and coping levels were compared in groups, as well as changes in clinical condition.

**Results:**

Migraine patients reported higher general loneliness and lower social support compared to healthy control subjects. Emotional loneliness was more marked in patients with higher headache impact. This subgroup of patients more frequently reported changes in the therapeutic and care paths as the perceived cause of the occurrence of motor or extra-motor symptomatology.

**Conclusions:**

Migraine patients, especially those more severely affected, proved more vulnerable than healthy control subjects to Covid-19 lockdown. Long-lasting interruption of social interactions may be detrimental in fragile patients that are in need of structured support interventions to maintain psycho-physical wellbeing.

## Introduction

Migraine is one of the more disabling neurological diseases, affecting more than 10% of general population (1). Clinically and nosographically migraine patients can be classified as episodic (EM) and chronic (CM), which crucially differ in terms of headache frequency (2). Chronic neurological patients are more vulnerable to sudden variations of habits, showing difficulties in adapting to stressors and acute events that alter the individual's functionality, worsen psycho-physical conditions – such as loneliness and distress – creating a more complicated response to treatment (3) and access to medical consultation (4). In times of crisis when people are forced into unexpected habit changes and uncertain life experience, pre-existing chronic conditions such as migraine, which is frequently associated to psychiatric comorbidity (5), requires particular attention as symptomatology can significantly worsen as a result of mood alterations and psychological vulnerability (6).

Recent literature provided controversial evidence about changes in clinical condition and symptomatology in migraine patients in the weeks after the Covid-19 outbreak. Parodi and colleagues (7) reported fewer headache attacks, lesser pain and moderate levels of depression in patients during the two-month quarantine. Delussi et al. (8) found an overall reduction in headache frequency and intensity during the quarantine, compared to pre-quarantine period, in relationship to the increased number of stay-at-home days and, as consequence, to reduced exposure to environmental factors that can exacerbate distress. At variance, other authors reported an overall negative impact of quarantine on patients, with an increased migraine frequency in the majority of interviewed patients (i.e., about 59.6%) and 10.3% of patients converting to chronic type (9). Marital status and sleep disorders have been suggested as possible influencing variables on headache attack frequency in migraine patients during Covid-19 pandemic (10). Also changes in eating habits and abuse in self-administered pharmacological therapies related to the subjective perception of clinical worsening contributed to modification of the psychological and physical status in migraine patients (11). Comparably, variations in migraine attacks have been reported in pediatric subjects undergoing online teaching (12). Overall, this evidence supports the role of individual variables in modulating migraine during the Covid-19 outbreak.

The spreading of Covid-19 pandemic caused a tremendous and drastic life change all over the world. Home confinement and social isolation became necessary to contain the incredible outbreak but a possible rebound of psychiatric disorders was announced by the scientific community. The PsyCovid study (13) underlined the importance of psychosocial variables in modulating individual reactions to the emergency in the Italian population. Individual perceptions of the emergency, indeed, appeared significantly modulated by psychosocial frailty (i.e., distress and loneliness), empathic skills and coping strategies (14). Comparably, other researchers have drawn the attention to the impact of Covid-19 pandemic on mental health in the general population (15), showing that women and young adults are more prone to suffer from post-traumatic stress disorders, depression, anxiety and sleep disturbances.

Social isolation and vulnerability in subjects bearing chronic diseases may prevent an efficient psychological adjustment and may negatively impact on symptom perception and individual adaptation to changes in life habits (16). Restrictive measures needed to contain Covid-19 spread and social distancing have been also particularly detrimental for those individuals in need of regular medical assistance, as migraine patients (9,17). In addition, persistent social isolation and psychological distress may affect the efficiency of the immune system (18), thus making individuals more prone to virus contagion.

In light of the above-mentioned evidence supporting the overall negative impact of restrictive measures in migraine individuals (9–11), the main aims of this study were: i) to explore individual psychosocial variables as loneliness and mood changes in migraine patients during the lockdown period that followed Covid-19 outbreak and ii) to explore the impact of Covid-19 related symptomatology and individual exposure to SARS-CoV2 contagion. Our main hypothesis was that higher levels of perceived isolation would correspond to higher headache impact, regardless of migraine subtype. In this frame, we anticipated that migraine type *per se* would not influence perceived changes in clinical condition, but migraine subjects with higher headache impact would report more detrimental changes in their clinical condition. As regards aim, our hypothesis was that perceived isolation was a more important driver of impact than Covid-19 exposure.

## Materials and methods

### Study design

This was a cross sectional survey conducted online in a group of subjects with migraine and in a group of healthy controls. The survey was implemented in Google Forms and distributed to eligible patients who formerly provided their informed consent to take part in the study, via written invitations through e-mails and Whatsapp. The online questionnaire included information about socio-demographic characteristics, psycho-socio-emotional variables, Covid-19 related symptomatology and SARS-CoV2 contagion risk, and ad hoc developed scales to assess perceived changes in clinical condition and related possible causes (see Measures paragraph for further details).

### Participants

Between 9 May and 2 June 2020, we carried out an online survey among the EM and CM patients belonging to the database of the Headache Center (a tertiary referral center) of Mondino Foundation IRCCS (Pavia, Italy). The database contains more than 4000 patients regularly followed at the Centre. All patients that had received a consultation within a 6-month period prior to the pandemic by a neurologist collecting migraine features and history were selected according to a simple random sampling method. Data were collected during the ‘Phase 2’ of the Italian lockdown post Covid-19 outbreak, when the Italian government imposed stringent containment measures that included restricted mobility, social distancing, need to wear face masks in outdoor and indoor spaces and adoption of remote activities whenever possible (e.g., smart working). The study protocol was approved by the local Ethics Committee of Mondino Foundation IRCCS (Pavia, Italy).

Eligibility criteria for patients with EM were i) age between 18 and 65 y.o., ii) the fulfillment of ICHD-3 criteria for migraine with or without aura, and iii) migraine duration ≥10 years. Patients with previous or present history of any other type of chronic headache (ICHD-3) were excluded from the EM group. Inclusion criteria for patients with CM were i) age between 18 and 65 y.o., ii) fulfillment of ICHD-3 criteria for CM, iii) no medication overuse. General exclusion criteria for both patient groups were the presence of i) dementia, ii) psychosis, and iii) intellectual disability.

Overall, 94 patients (i.e. 56 CM and 38 EM) were contacted by phone and invited to complete the survey online. The response rate was 89.36% (chronic 92.5% and episodic 84.21%), calculated as the ratio of the number of actual responders to the total number of patients invited to the study. Non-responders were patients who did not provide their informed consent to participate or who did not complete the survey. A final set of 84 patients (i.e. 52 CM (62% of the sample; 44 females, mean age = 48.38, sd = ±10.70) and 32 EM (38% of the sample; 27 females, mean age = 37.88, sd = ±12.48) completed the survey.

According to the study hypothesis, in which we also aimed to assess possible differential effect of Covid-19 pandemic in episodic *vs.* chronic migraine patients, we sub-grouped the whole patient sample comparing EM patients (n = 32) with a number of gender, age and education matched CM group of patients (n=32).

Additionally, we used a classification procedure – i.e., Two Step Cluster Analysis – to split the whole sample into different clusters on the basis of the Headache Impact Test scale (HIT-6) (19), regardless of the type of migraine. A major advantage of this classification approach is related to the determination of the number of clusters, which is not grounded on an arbitrary choice like more traditional clustering techniques, but rather relies on a statistical measure of fit (e.g., *Bayesian information criterion* or BIC, like in our case). As a result we obtained two clusters according to the HIT-6 score: a high headache impact group (HHI; n = 38, 31 females, mean age = 40.16, sd = ±11.48) and a low headache impact group (LHI; n = 26, 23 females, mean age = 40.12, sd = ±10.98). This resulting membership variable was used as grouping factor in further statistical analysis.

Finally, we selected a group of 64 matched healthy controls (HC; 54 females, mean age = 40.03, sd = ±11.22) from the PsyCOVID Study dataset (14,20) for statistical comparison with migraine patients. Inclusion criteria for HC were i) age between 18 and 65 y.o., ii) absence of any neuropsychiatric disorder, iii) no intake of psychoactive medications, and iv) negative history for migraine, frequent or chronic tension-type headache.

### Measures

#### Psycho-socio-emotional measures

We assessed psycho-social-emotional dimensions as they have a crucial role in emergency settings and crisis situations. These dimensions included perceived global distress (21), loneliness (21), empathic skills (22), and coping strategies (23). We assessed global distress with the Italian version of the Depression Anxiety Stress Scales-21 (DASS-21) (24), which allows to calculate three specific scores related to distress, anxiety and depression levels. We used two sub-scales of the Interpersonal Reactivity Index (IRI) (25) to assess emotional (Empathic Concern sub-scale) and cognitive (Perspective-Taking sub-scale) components of empathic abilities that have been proved to be highly reliable in detecting empathic attitude during crisis time as Covid-19 pandemic (26). We finally assessed three different facets of loneliness with the Italian Loneliness Scale: General Loneliness, Social Loneliness, and Emotional Loneliness (27). The three scales explore respectively i) a global measure of loneliness, conceptualized as the significant reduction/lack of interpersonal relationship; ii) the (perceived) lack of a supportive social network (i.e., friends, people who give help when necessary); iii) the (perceived) lack of significant/desired relations, related to experiences of emotional abandonment (27). Coping styles were assessed with the short version of the Italian version of the Coping Orientation to the Problems Experienced (COPE-NVI-25) (28), measuring diverse coping behaviors/styles towards problems or stressful events (Positive attitude, Problem orientation, Transcendence orientation, Social support, Avoidance strategies).

#### Covid-19 related symptomatology and individual exposure to the SARS-CoV-2 contagion

Then, we evaluated Covid-19 related symptomatology and individual risk of the SARS-CoV-2 contagion by means of a multidimensional score assessing Covid-19 risk profile. We considered a total of 10 variables, representing living area/region (e.g., big vs. small city), Covid-19 major symptoms and face-to-face contacts with others (see Appendix A for the complete list of variables). For each item, we assigned a score 0 in the absence and 1 in the presence of the status/condition. The Covid-19 score was then calculated by dividing the sum of the deficits presented by each participant by the total number of variables measured.

#### Perceived changes in clinical condition and possible causes

We finally assessed individual perceived changes in clinical condition after the Covid-19 outbreak and the consequential lockdown. First, we asked patients to rate on a 5-point Likert scale how much (0 = Not at all – 4 = Extremely) the imposed restrictive measures to contain Covid-19 spread changed their clinical condition with the appearance of motor (e.g., stress resistance, stiffness, fatigue) or extra-motor (e.g. intestinal or urinary disorders, sleep disturbances, eating disorders, difficulty in concentration, irritability) symptomatology.

Then, we asked patients to rate on a 5-point Likert scale how much (0 = Not at all – 4 = Extremely) their motor and extra-motor symptom changes, if any, were due to modifications in: i) therapeutic and care paths (e.g., difficulty in obtaining prescriptions/medications, inability to carry out the planned checkups/recoveries, difficulty in contacting the treating specialist), ii) interpersonal relationship with cohabitants (e.g., tensions with family members, inability to see non-cohabiting family members), iii) drop of social relationships (e.g., social and recreational activities reduction), and iv) decrease of physical activity (e.g., inability to perform physiotherapy and motor rehabilitation, inability to exercise or to go to the gym).

### Statistical analysis

We carried out statistical analyses using SPSS (https://www.spss.it/). We set statistical significance at p < 0.05 for all statistical tests we performed. We calculated descriptive statistics including frequencies and percentages for categorical variables, and mean and standard deviation for pseudo-continuous variables. Preliminarily, we explored the distribution for each variable with the Kolmogorov–Smirnov test. Although some variables did not show a normal distribution, we analyzed data by testing parametric models as well (one-way ANOVA, mixed ANOVA) according to the results from Blanca et al. (29).

We first used one-way ANOVA to compare patient groups, classified either to migraine subtype or headache impact, with the HC group (i.e., *Analysis 1*: EM *vs.* CM *vs.* HC; *Analysis 2*: HHI *vs.* LHI *vs.* HC) on variables related to stress, depression and anxiety, empathic abilities, loneliness and coping styles (see Psycho-socio-emotional measures paragraph). We used the Bonferroni correction to adjust results for multiple comparisons (corrected p-value = 0.004). Then, we compared patient groups and HC (one-way ANOVA; *Analysis 3*: EM *vs.* CM *vs.* HC; *Analysis 4*: HHI *vs.* LHI *vs.* HC) on the Covid-19 risk profile score, that merges information related Covid-19 symptomatology and individual risk of the SARS-CoV-2 contagion.

Finally, we compared scores reporting changes in clinical condition and possible causes between patient groups, classified either to migraine subtype (EM and CM) or headache impact (HHI and LHI). Specifically, we computed (a) a Chi-square Test of Independence on a set of four 2x2 contingency tables, considering patient groups (EM and CM; HHI and LHI) and presence/absence (presence: rating = 1-4; absence: rating = 0) of motor or extra-motor symptom changes and (b) a Chi-square Test of Independence on a set of four 2x5 contingency tables, considering patient groups (EM *vs.* CM; HHI *vs.* LHI) and the degree of perceived motor or extra-motor symptom changes.

We performed the same analytic procedure to explore possible causes for perceived changes in clinical condition. In detail, we computed a Chi-square Test of Independence on a set of sixteen 2x2 contingency tables, considering patient groups (EM and CM; HHI and LHI) and presence/absence (presence: rating = 1-4; absence: rating = 0) of changes in therapeutic/assistance path, interpersonal relationship with cohabitants, drop of social relationships, and decrease of physical activity. Additionally, we performed a Chi-square Test of Independence on a set of sixteen 2x5 contingency tables, considering patient groups (EM *vs.* CM; HHI *vs.* LHI) and the rating distribution of causes attributed by the patients to perceived changes in clinical condition.

## Results

### Demographic and socio-economic information on the sample

Demographic and socio-economic information of final set of migraine patients, classified according to migraine type or headache impact, and control subjects are reported in Table 1.

**Table 1.** Descriptive socio-demographic characteristics of migraine patients and control subjects.

	CM	EM	HC	Statistics
*Number of subjects (male : female)*	32 (5:27)	32 (5:27)	64 (10:54)	*÷*²(2) = 0.001; *p* = 1.000
*Age in years (mean ± st.dev)*	42.41 ± 9.39	37.88 ± 12.48	40.03 ± 11.22	*F*(2, 125) = 1.326 *p* = 0.269
*Education in years (mean ± st.dev)*	13.50 ± 3.40	14.44 ± 2.85	14.20 ± 2.72	*F*(2, 125) = 0.915; *p* = 0.403
*Civil Status (In a relationship:Other)*	18:14	15:17	32:32	*÷*²(2) = 0.594; *p* = 0.743
*Working Status (Employed:Unemployed/Retired)*	27:4	29:3	54:10	*÷*²(2) = 0.727; *p* = 0.695
*Living condition (n° inhabitants, mean ± st.dev)*	2.94 ± 1.34	3.19 ± 1.30	2.72 ± 1.35	*F*(2, 125) = 1.32; *p* = 0.270
	HHI	LHI	HC	Statistics
*Number of subjects (male : female)*	38 (7:31)	26 (3:23)	64 (10:54)	*÷*²(2) = 0.555; *p* = 0.758
*Age in years (mean ± st.dev)*	40.16 ± 11.48	40.12 ± 10.98	40.03 ± 11.22	*F*(2, 125) = 0.002 *p* = 0.998
*Education in years (mean ± st.dev)*	14.03 ± 3.01	13.88 ± 3.39	14.20 ± 2.72	*F*(2, 249) = 0.056 *p* = 0.945
*Civil Status (In a relationship:Other)*	17:21	16:10	32:32	*÷*²(2) = 1.775; *p* = 0.412
*Working Status (Employed:Unemployed/Retired)*	33:4	23:3	54:10	*÷*²(2) = 0.565; *p* = 0.754
*Living condition (n° inhabitants, mean ± st.dev)*	2.89 ± 1.33	3.31 ± 1.32	2.72 ± 1.35	*F*(2, 249) = 0.691 *p* = 0.502

CM: chronic migraine; EM: episodic migraine; HC: healthy control subjects; HHI: high headache impact; LHI: low headache impact.

### Group comparisons on psycho-emotional-social variables

The two one-way ANOVAs performed to compare patient groups and HC (*Analysis 1*: CM *vs.* EM *vs.* HC; *Analysis 2*: HHI vs. LHI vs. HC) showed similar results. In both cases, we found significant differences among groups in the three loneliness dimensions assessed, namely General Loneliness (*Analysis 1*: *F*(2,125) = 19.398, *p* < 0.001; *Analysis 2*: *F*(2,125) = 20.174, *p* < 0.001), Social Loneliness (*Analysis 1*: *F*(2,125) = 13.668, *p* < 0.001; *Analysis 2*: *F*(2,125) = 14.556, *p* < 0.001) and Emotional Loneliness (Analysis 1: *F*(2,125) = 18.474, *p* < 0.001; Analysis 2: *F*(2,125) = 21.287, *p* < 0.001). We did not find significant group differences on the other psycho-socio-emotional variables.

Concerning *Analysis 1* (CM *vs.* EM *vs.* HC), post-hoc analysis revealed that both patient groups presented significantly higher scores than HC in General and Emotional Loneliness – i.e., patients with migraine felt more lonely than HC (*p*_GL_ < 0.001; *p*_EL_ < 0.001) – and lower scores in Social Loneliness than HC (*p*_SL_ < 0.001) - i.e., patients with migraine felt themselves as having a poorer social support than HC. We did not detect any difference between CM and EM (*p*_EL_ = 0.59; *p*_GL_ = 0.91; *p*_SL_ = 1.00).

Concerning *Analysis 2* (HHI *vs.* LHI *vs.* HC), post-hoc analysis showed that patient groups presented significantly higher scores than HC in General Loneliness (*p*_GL_ < 0.001), and significantly lower scores than HC in Social Loneliness (*p*_SL_ < 0.001), but we found no significant differences between HHI and LHI (*p*_GL_ = 0.41, *p*_SL_ = 0.69). Emotional Loneliness scores, instead, significantly differentiated the three groups, with HHI reporting significantly higher scores compared to LHI (*p* = 0.045) and to HC (*p* < 0.001), and LHI presenting higher scores than HC (*p* = 0.010).

See Table 2 for details on psycho-emotional-social variables in groups.

**Table 2.** Descriptive features of psychosocial variables in migraine patients and control subjects.

	CM (n = 32)	EM (n = 32)	HC	Statistics	Post Hoc
*DASS-21 Anxiety*	2.44 ± 2.69	2.41 ± 3.20	2.27 ± 3.02	F(2, 125) = 0.042 p = 0.959	–
*DASS-21 Stress*	6.38 ± 4.50	5.16 ± 4.03	6.06 ± 4.65	F(2, 125) = 0.665 p = 0.516	–
*DASS-21 Depression*	4.75 ± 4.69	2.59 ± 2.79	3.83 ± 3.93	F(2, 125) = 2.469 p = 0.089	–
*ILS Social Loneliness*	9.37 ± 3.25	9.40 ± 3.75	13.14 ± 4.53	F(2, 125) = 13.668 p < 0.001	CM < HC; EM < HC
*ILS Emotional Loneliness*	12.03 ± 4.54	10.69 ± 2.95	7.03 ± 4.40	F(2, 125) = 18.474 p < 0.001	CM > HC; EM > HC
*ILS General Loneliness*	13.25 ± 4.81	12.06 ± 3.71	7.66 ± 4.88	F(2, 125) = 19.398 p < 0.001	CM > HC; EM > HC
*IRI Perspective Taking*	17.66 ± 5.35	16.78 ± 4.50	18.19 ± 4.03	F(2, 125) = 1.040 p = 0.356	–
*IRI Emotional Concern*	20.72 ± 4.03	20.03 ± 4.11	20.97 ± 3.98	F(2, 125) = 0.580 p = 0.562	–
*COPE Positive Attitude*	21.88 ± 6.04	21.88 ± 5.27	23.39 ± 5.31	F(2, 125) = 1.216 p = 0.300	–
*COPE Social Support*	18.38 ± 5.69	18.16 ± 4,15	19.47 ± 5.35	F(2, 125) = 0.879 p = 0.418	–
*COPE Problem Orientation*	21.13 ± 5.66	19.81 ± 4.16	20.58 ± 4.52	F(2, 125) = 0.618 p = 0.540	–
*COPE Transcendence Orientation*	9,44 ± 5.96	9.81 ± 5.80	9,47 ± 6.29	F(2, 125) = 0.041 p = 0.960	–
*COPE Avoidance Strategies*	10.44 ± 3.73	9,81 ± 3.20	9.52 ± 3.12	F(2, 125) = 0.830 p = 0.438	–
	HHI (n = 38)	LHI (n = 26)	HC	Statistics	
*DASS-21 Anxiety*	2.71 ± 3.08	2.00 ± 2.71	2.27 ± 3.02	F(2, 125) = 0.453 p = 0.637	–
*DASS-21 Stress*	6.76 ± 4.71	4.31 ± 3.12	6.06 ± 4.65	F(2, 125) = 2.468 p = 0.089	–
*DASS-21 Depression*	4.58 ± 4.57	2.35 ± 2.44	3.83 ± 3.93	F(2, 125) = 2.557 p = 0.081	–
*ILS Social Loneliness*	9.89 ± 3.62	8.65 ± 3.19	13.14 ± 4.53	F(2, 125) = 14.556 p < 0.001	HHI < HC; LHI < HC
*ILS Emotional Loneliness*	12.39 ± 4.04	9.85 ± 2.88	7.03 ± 4.40	F(2, 125) = 21.287 p < 0.001	HHI > HC; LHI >HC; HHI < LHI
*ILS General Loneliness*	13.37 ± 4.79	11.62 ± 3.31	7.66 ± 4.88	F(2, 125) = 20.174 p < 0.001	HHI > HC; LHI > HC
*IRI Perspective Taking*	17.29 ± 5.00	17.12 ± 4.90	18.19 ± 4.03	F(2, 125) = 0.747 p = 0.476	–
*IRI Emotional Concern*	20.42 ± 4.16	20.31 ± 3.97	20.97 ± 3.98	F(2, 125) = 0.352 p = 0.704	–
*COPE Positive Attitude*	22.71 ± 6.00	20.65 ± 4.88	23.39 ± 5.31	F(2, 125) = 2.337 p = 0.101	–
*COPE Social Support*	18.42 ± 5.44	18.04 ± 4.20	19.47 ± 5.35	F(2, 125) = 0.907 p = 0.406	–
*COPE Problem Orientation*	21.00 ± 4.46	19.69 ± 5.64	20.58 ± 4.52	F(2, 125) = 0.592 p = 0.554	–
*COPE Transcendence Orientation*	9.71 ± 6.07	9.50 ± 5.60	9,47 ± 6.29	F(2, 125) = 0.020 p = 0.980	–
*COPE Avoidance Strategies*	10.53 ± 3.83	9.54 ± 2.81	9.52 ± 3.12	F(2, 125) = 1.420 p = 0.292	–

CM: chronic migraine; EM: episodic migraine; HC: healthy control subjects; HHI: high headache impact; LHI: low headache impact; DASS-21: Depression Anxiety Stress Scales-21; ILS: Italian Loneliness Scale; IRI: Interpersonal Reactivity Index; COPE: Coping Orientation to the Problems Experienced.

### Comparisons on the Covid-19 risk profile score

Neither of the analyses performed (*Analysis 3:* EM *vs.* CM *vs.* HC; *Analysis 4:* HHI *vs.* LHI *vs.* HC) revealed significant results (*Analysis 3: F*(2,125) = 1.630, *p* = 0.20; *Analysis 4: F*(2,125) = 2.594, *p* = 0.079), indicating that on average patients and HC did not differ in terms of Covid-19 related symptomatology or individual risk to SARS-CoV-2 contagion.

Differences in perceived changes in clinical condition and possible causes

Overall, the majority of patients reported changes in both motor (50% to 69%) and extra-motor (69% to 76%) symptoms. Such percentages did not differ when considering either migraine type (i.e., EM or CM; *motor*: *X*^2^(1) = 2.33, *p* = 0.127; *extra-motor*: *X*^2^(1) = 0.08, *p* = 0.777) or headache impact (i.e., HHI and LHI; *motor*: *X*^2^(1) = 0.55, *p* = 0.456; *extra-motor*: *X*^2^(1) = 0.39, *p* = 0.529) as grouping variables. Comparably, the degree of perceived motor and extra-motor changes was similar in patients with different migraine types (i.e., EM *vs.* CM; *motor*: *X*^2^ (4) =  4.74, *p* = 0.346; *extra-motor*: *X*^2^ (4) = 5.99, *p* = 0.20) or headache impacts (i.e., HHI *vs.* LHI; *motor*: *X*^2^(4) = 0.77, *p* = 0.943; *extra-motor*: *X*^2^(4) = 1.73, *p* = 0.786).

Statistical analysis showed significant differences between headache impact groups, but not between migraine types, in variables assessing possible causes of perceived changes in clinical condition. In particular, we found that a larger proportion of HHI compared to LHI patients reported changes in the therapeutic and care paths as the cause for both motor (*X*^2^(1) = 5.92, *p* = 0.015) and extra-motor (*X*^2^(1) = 4.04, *p* = 0.045) changes.

Additionally, we observed that a larger proportion of HHI compared to LHI patients indicated the decrease of physical activity as a possible cause of the worsening of their motor symptomatology (*X*^2^(1) = 6.46, *p* = 0.011). The degree of physical activity decrease as cause of motor changes showed significantly different result with regard of the headache impact sub-grouping (*X*^2^(4) = 10.46, *p* = 0.033). A post-hoc analysis based upon standardized residuals indeed showed that HHI patients significantly differed from LHI patients (*X*^2^(1) = 5.29, *p* = 0.02) in the distribution of the ‘extremely’ rating point, selected by 19% of HHI patients and by none of the LHI patients.

Numbers and frequency of perceived motor and extra-motor changes in clinical condition and possible related causes are reported in Table 3.

**Table 3.** Motor and extra-motor changes in clinical condition and related causes in patient groups.

	Migraine type		Headache impact	
	CM (n = 32)	EM (n = 32)	HHI (n = 38)	LHI (n = 26)
***Perceived changes in clinical condition***				
Number of patients with motor changes *(percentage of frequency)*	22 (69%)	16 (50%)	24 (63%)	14 (54%)
Number of patients with extra-motor changes *(percentage of frequency)*	23 (72%)	24 (75%)	29 (76%)	18 (69%)
***Possible causes of motor changes***				
Therapeutic/care paths *(percentage of frequency)*	8 (26%)	7 (24%)	**13 (36%)**	**2 (8%)**
Interpersonal relationship with cohabitants *(percentage of frequency)*	17 (55%)	14 (48%)	20 (56%)	11 (46%)
Drop of social relationships *(percentage of frequency)*	21 (68%)	17 (59%)	24 (67%)	14 (58%)
Decrease in physical activity *(percentage of frequency)*	21 (68%)	18 (62%)	**28 (78%)**	**11 (46%)**
***Possible causes of extra-motor changes***				
Therapeutic/care paths *(percentage of frequency)*	10 (35%)	6 (21%)	**13 (37%)**	**3 (13%)**
Interpersonal relationship with cohabitants *(percentage of frequency)*	16 (53%)	16 (53%)	19 (54%)	13 (54%)
Drop of social relationships *(percentage of frequency)*	20 (67%)	18 (64%)	24 (69%)	14 (61%)
Decrease in physical activity *(percentage of frequency)*	15 (52%)	18 (62%)	22 (63%)	11 (48%)

## Discussion

Migraine is considered one of the most disabling chronic disorder due to its early onset and its impact on different dimensions of patients’ life (30). The impact of this neurological condition can be worsened by critical and stressing situations threatening individual psycho-physical integrity. Stress is indeed one of the most common headache trigger factors, able to increase headache frequency and promote chronicization (31), and migraine patients, in particular those with a chronic profile, seem to be less able to cope and tolerate stress than healthy controls (32). In this frame, Covid-19 emergency, causing large reorganization of private and public life in people, represented, and still does, a critical period for every individual. In this time, patients suffering from a chronic condition, such as migraine, are thus more at risk to experience worsening in their clinical condition (9) and experience more severe psychological distress that general population (33).

Our study is the first to specifically address psychosocial variables in a representative sample of migraine patients during the lockdown period using a thorough approach based on multiple validated tools to explore different psychosocial dimensions. In particular, we showed that patients with chronic and episodic migraines reported higher levels of general and emotional loneliness and lower levels of perceived social support (i.e., social loneliness) than controls, despite the absence of significant differences in the individual exposure to the SARS-CoV-2 contagion among groups. A similar pattern emerged also in General and Social Loneliness variables when we grouped patients on the basis of the self-reported headache impact on everyday life (HIT-6 score). However, HHI and LHI patients were discriminated by the Emotional Loneliness scores. Emotional loneliness that is prompted by the subjective evaluation of the lack of desired interpersonal relationships has proved more damaging for health (34) and relevant to mortality in older adults (35), compared to the effects possibly exerted by social loneliness, which instead arises as a result of the perception of the lack of a supportive social network. Moreover, as recently reported, loneliness has a crucial impact on illness experience in patients with migraine, particularly in the chronic form, reflected in the reduced ability in self-management and the individual satisfaction towards the current state of care (36,37). The investigation of this psychosocial dimension in crisis time may offer crucial information about differential disease courses and treatment failures in individuals with migraine.

These findings support the importance of psychosocial variables as relevant modulators of disease condition during Covid-19 pandemic. In particular, we identified loneliness as a key psychosocial variable in migraine patients – affecting both episodic and chronic patients’ groups – in the post Covid-19 outbreak time. This result is in line with the evidence of higher migraine attack frequency in divorced individuals during the Covid-19 pandemic, as proved by Ma et al. (10). Generally, both cognitive and physical statuses can influence the individual perception of loneliness (27) and the experience of a chronic illness is often associated with the presence of a psychological state of loneliness (e.g., 36,37). Extraordinary life events, such the social isolation imposed during the lockdown, can magnify this state of loneliness (27).

The early detection of psychosocial vulnerability following the Covid-19 outbreak in fragile categories may prevent long-lasting health status changes, and would prove helpful in the future to prevent consequences on general well-being by allocating resources to support targeted interventions to manage psychosocial distress and increase young adult and elderly resilience towards the post-Covid-19 crisis. Long-term strategies should thus be validated and implemented to deliver quality care for patients with migraine, with emphasis on psychosocial well-being. Paying particular attention to marital or family status (e.g. being alone at home or not) should be recommended in order to better support more vulnerable subjects and implement an individualized strategy to support them during crisis time.

Finally, as previously reported (9,17), the majority of migraine patients here enrolled experienced problems in maintaining their usual therapeutic and care path (e.g., difficulties to communicate with their neurologist or to receive their periodic botox injections) due to Covid-19 pandemic. Indeed, even if we failed to detect differences in episodic *vs.* chronic migraine patients in the perception of motor and/or extra-motor changes in clinical condition, patients referring a higher impact of headache on everyday life (HHI group) reported more frequently changes in the therapeutic and/or care path as a main reason of such clinical changes.

The major limitations of the present study are represented by the cross-sectional nature of the study, the small-to-moderate sample size, the use of a convenience sampling and of self-reported measures to evaluate psychosocial dimensions, this latter due to the pandemic that prevented us from directly collecting patient data. Further longitudinal study in migraine patients could allow drawing more strong causal conclusions on individual trajectories of psychosocial malaise in episodic and chronic patients. Preliminary findings from our work and previous studies (38) collected during the Covid-19 pandemic support the worry about a possible exacerbation of pre-existent psycho-physical disorders in chronic patients, including migraine. Restrictive measures induced by the Covid-19 pandemic in fact had a high impact on patients, causing isolation, restrictions on movements, impoverishment of social contacts and affective relationships, increased perception of loneliness, and forcing changes in habits and routines.

In conclusion, clinicians and the scientific community should be aware of the need to identify alternative solutions to overcome social isolation, treatment discontinuations and related risks in migraine. The adoption of different strategies, such as telemedicine, online follow-up for treatment, online tools to assess both physical and psychological symptoms, may prove useful because of their ability to easily overcome safety and distancing problems, without renouncing the effectiveness of treatments (39,40). During this pandemic experience some attempts have been made in this direction (41), but surely more effort and resources need to be implemented to create alternative clinical strategies and pathways that allow patients to access to medical and psychological support even in dramatic conditions.

## Clinical implications

1. Migraine patients reported higher general loneliness and lower social support compared to HC in the weeks after Covid-19 lockdown.
2. Emotional loneliness was more marked in patients with higher headache impact.
3. Patients with higher headache impact reported more frequently changes in the therapeutic and care paths as the perceived cause of the occurrence of motor or extra-motor symptomatology.
4. Migraine patients, especially those more severely affected, proved more vulnerable than HC to Covid-19 lockdown.
5. Long-lasting reduction of social interactions may be detrimental in fragile patients that would need of structured support interventions to maintain psycho-physical wellbeing.

## Appendix A.

The 10 items included in the *Covid-19 Index.*

**Table:**

*Living area*	
1	Resident in Northern Italy
2	Resident in a big/metropolitan city
*Covid-19 related symptoms*	
3	Actual presence of fever
4	Actual presence of dyspnea
5	Actual presence of cough and/or cold
6	Actual presence of physical malaise/smell or taste disorders not otherwise explainable
*Face-to-face contacts with…*	
7	A suspect case of SARS-CoV2 (≥ once in a week)
8	An asymptomatic case of SARS-CoV2 (≥ once in a week)
9	A symptomatic case of SARS-CoV2 (≥ once in a week)
10	More than five people a day during the lockdown (from 2020, March 9th, to the date of survey completion)
